# Flipping the Lens: Using Instagram for Photovoice and Videovoice With LGBTQ+ Migrants in São Paulo

**DOI:** 10.1177/10778004261417671

**Published:** 2026-02-05

**Authors:** Tyler Valiquette, Yvonne Su

**Affiliations:** 1 University College London, UK; 2 York University, Toronto, Ontario, Canada

**Keywords:** Instagram, photovoice, videovoice, LGBTQ+ refugees, intersectionality, South–South migration

## Abstract

This article examines Instagram as a tool for data collection with LGBTQ+ refugees and migrants in São Paulo, Brazil. While photovoice and videovoice are widely used, integrating Instagram enables forms of data collection not usually possible with these methods. Twenty-one LGBTQ+ migrants created original visual content. Instagram allowed participants creative control compared with conventional photovoice, choosing how to represent themselves through filters, captions, music, and privacy settings, while negotiating constraints such as digital literacy and platform visibility. Drawing on workshops, content, and interviews, the article analyzes how Instagram facilitated storytelling, community building, and agency, while highlighting the limits of digital participation.

## Introduction

This article positions itself as a methodological reflection on the use of photovoice and videovoice in queer migration research, with a specific focus on Instagram as an underutilized but highly effective participatory platform. Drawing on an empirical study with LGBTQ+ refugees and migrants in São Paulo, Brazil, it demonstrates how Instagram can function not only as a dissemination tool but as a research tool for data collection that advances co-production and participant agency. The integration of the platform in the methods of photovoice and videovoice allows for collecting data in ways that are not usually done when applying these visual participatory methods. Alongside its methodological focus, the article also engages with the ethical and theoretical questions raised by digital participatory research, such as how do we balance letting participants take ownership of their stories with ethical concerns over consent, surveillance and platform control? And theoretically, do digital tools like Instagram allow for more intersectional representations of participants than traditional photovoice and videovoice methods? Participatory methods have gained academic praise for facilitating the co-production of knowledge in a collaborative manner that encourages “exchange” versus “extraction” (Marzi, 2021). Yet within queer migration scholarship, where visual and participatory approaches remain underutilized, Instagram offers a distinctive methodological innovation by aligning data creation with participants’ everyday practices of digital self-expression. Specifically, regarding refugee-focused research, participatory approaches help reduce the power imbalance between researcher and participant. Photovoice and videovoice allow LGBTQ+ asylum seekers, refugees, and migrants to present their world to the global community by showcasing their needs, hopes, and priorities.

We used both photovoice and videovoice because using them together allows for more creative freedom and expression in the type of content participants produce. The two approaches also allow for more agencies in storytelling by providing multiple avenues for sharing, reflection, and expression (Mitchell et al., 2017; Valiquette & Su, 2024). Using Instagram as a research tool allows participants to express their creativity through writing captions, adding music to videos, including hashtags, or following viral trends. We argue that this approach expanded creative control through captions, filters, music, and privacy settings, while reducing researcher intervention and aligning knowledge production with participants’ existing media practices. Traditional videovoice and photovoice methods can often be overly prescriptive, imposing specific guidelines that reflect the researcher’s preferences rather than allowing for more flexible, participant-driven approaches (Castleden et al., 2008; Seitz & Orsini, 2022). Our approach promotes more free-thinking by encouraging participants to tell their stories of migration, asylum-seeking, and placemaking in their unique style and voice, and centers the knowledge and experiences of the affected people recognizes the complexities of their diverse social identities. It also serves as a platform for social mobilization, challenging the reduction of queer migrants and refugees to passive statistics and highlighting their agency and multifaceted identities.

This article explores how Instagram serves as a data collection tool in photovoice and videovoice methods, facilitating the co-production of knowledge with LGBTQ+ refugee and migrant communities. We draw on the Instagram photos and videos created by 21 LGBTQ+ refugees and migrants in São Paulo, Brazil, from February to July 2023, the conversations during the training workshops, and the follow-up interviews and reflections with the participants. The Instagram-based component is a central focus, and we evaluate how participants’ choices in posting, captioning, and sharing shaped the research process of their narratives. Building on the participants’ experiences, we examine how photovoice, videovoice, and digital media can empower vulnerable and mobile communities, enhancing their sense of agency in the research process. Furthermore, we discuss how applying an intersectional lens to participatory methods makes it possible to design data collection in ways that are more sensitive to how participants share their multiple social positions, better capturing their experiences of migration and mobility. This article contributes directly to queer migration scholarship by focusing on the lived experiences of gender and sexual minority migrants and refugees, revealing “the indeterminacy, contingency, malleability, and often oppressive nature” of normative binary systems on queer mobility (Knopp & Brown, 2003).

## Photovoice and Videovoice as Creative Methods

Photovoice, a participatory method, seeks to redress power imbalances between researchers and participants by enabling co-researchers to document and reflect on their lived experiences through visual media (Wang & Burris, 1997). Initially used in health research, it has since become a widely adopted arts-based method for engaging marginalized groups (Adinia & Kirana, 2019; Miled, 2020; Teti et al., 2021). By encouraging critical reflection and dialogue around participants’ images, photovoice fosters agency, pride, and ownership while challenging narratives that frame marginalized people as passive and vulnerable (Mitchell et al., 2017; Sutton-Brown, 2014). These methods also drive policy and advocacy efforts toward social justice that the participants themselves play a role in shaping (Hill Collins, 2019; Kurtiş & Adams, 2017).

Videovoice expands on these participatory and reflexive principles through motion, sound, and narration, providing richer layers of representation and emotional depth (Catalani et al., 2012; Latz & Mulvihill, 2017). Participants use cameras or phones to create short films that document their daily lives and social realities, transforming data collection into a form of advocacy and storytelling. Similar to photovoice, videovoice has been primarily used in health-related research and cases related to health and disaster studies. Like photovoice, videovoice is praised for empowering participants and producing compelling narratives for social change but is also critiqued for its reliance on technical access, editing mediation, and potential overexposure of participants’ private lives (Milne et al., 2012). Together, these participatory visual methods blur the boundaries between research, art, and activism, offering powerful means of expression while demanding reflexivity about representation, authorship, and impact.

A growing body of migration studies and mobility research has emerged in which arts-based methods, including photovoice, were used as a participatory method with refugees and asylum seekers (Adinia & Kirana, 2019; Miled, 2020). Videovoice has been employed in various research studies to empower communities, assess community needs, and promote social change (Blumenstock et al., 2015). Despite photovoice gaining popularity as a research approach with refugee populations, few studies (Valiquette & Su, 2024) exist where it is used with LGBTQ+ refugees. The critiques of photovoice and videovoice have focused on the equalizing power relationships between researcher and participant, and despite these methods offering potential to amplify the voices of marginalized communities, power relationships between researcher and participant can remain unbalanced (Marzi, 2021). Feminist critiques of these methods highlight how they are often framed around notions of empowerment, implying that refugee and migrant communities lack agency and need researchers to “give them a voice,” as though they do not already have one (Logan & Murdie, 2016). Another tension concerns the pressure to satisfy the academic output requirements of the research process, particularly concerning timelines and expected deliverables (Marzi, 2021). Consequently, researchers may interfere with the filming and editing processes, thereby challenging the agency of participants in how their stories are developed and represented (Mistry & Berardi, 2012). These critiques highlight a mismatch between the theory and the practice of participatory research, reinforcing traditional power hierarchies which photovoice and videovoice are argued to challenge (Marzi, 2021; Shaw, 2016).

## Photovoice and Videovoice: The Role of Intersectionality

It is important to consider intersectionality in our research approach and how issues of class, gender, disability, educational background, citizenship, sexuality, or age affect perspectives, given that our participants come from diverse backgrounds (Nasser-Eddin & Abu-Assab, 2020). Intersectionality was initially construed as a means for exploring and addressing overlapping experiences of oppression faced by African American women through the intersections of race and gender, facing institutionalized marginalization (Crenshaw, 1989). Since then, the concept has developed and generated significant engagement and debate as a key framework for analyzing power, identity, and marginalization across academic disciplines (Hill Collins, 2019; Lenette, 2020; Romero, 2017). Intersectional analysis simultaneously involves the importance of multiple and interconnected social categories, such as race and the processes of racialization, gender, sexuality, and age, as being mutually constitutive as opposed to independent markers of identity (Fiddian-Qasmiyeh, 2020). A careful focus must be paid to diverse and overlapping power structures and precarities related to multiple and interconnected social categories that create marginalization, exploitation, and violence. To fully capture the lived experiences of marginalized communities, migration researchers must look beyond solely gender and sexuality to also consider other intersecting social categories such as age, race, indigeneity, class, disability, education, visa status, socioeconomic status, ethnicity, religion, and citizenship (Lenette, 2020).

In migration and mobility studies, intersectional approaches highlight how experiences of displacement, seeking refuge, and forced migration are shaped not only by identity markers but also by power structures such as racism, xenophobia, homophobia, and transphobia (Fiddian-Qasmiyeh, 2020; Su et al., 2021). These power structures operate dynamically, interacting with one another and shifting based on social, cultural, and political contexts. The relative importance of these identity markers and the power structures they reflect evolves across time and space, particularly in displacement contexts, highlighting that vulnerability is shaped by context rather than fixed to specific identity “categories” (Crawley & Kleparis, 2018; Fiddian-Qasmiyeh, 2020). For instance, while a displaced individual may face xenophobia in one country, their identity as a queer person or as a woman may become more or less significant in shaping their experiences in another. In research focused on LGBTQI+ migrants and refugees, there is a tendency to focus specifically on experiences of violence and discrimination, implying a narrative of victimhood (Camminga & Marnell, 2022). These depictions mirror broader debates that perceive refugees as passive, helpless, and vulnerable (Ongwech et al., 2024). There has been little attention to how forced migrants with diverse sexual and gender identities actively navigate and make sense of their intersecting identities, with only a few notable contributions from queer migration scholarship beginning to address this gap.

The literature on intersectionality in relation to participatory methods dispels the misconception that the lived experiences of marginalization and discrimination can be easily captured (Lenette, 2020). An intersectional lens applied to methodologies allows for exploring multiple and shifting identities instead of focusing on one aspect and excluding other narratives (Lenette, 2020; Nasser-Eddin & Abu-Assab, 2020). When photovoice and videovoice are analyzed through an intersectional lens, these narratives highlight the ways participants’ social identities intersect in experiences of marginalization and violence. These methods provide a platform for storytelling and advance research and practice by identifying tangible points for intervention (Teti et al., 2021).

## Enhancing Migrant Agency: Using Digital Tools in Photovoice and Videovoice Research

Focusing on migrant agency is critical in understanding how policymakers, activists, and academics attempt to understand, theorize, and design policies and campaigns concerning migration. Agency refers to people’s opportunities and capabilities to exercise power, negotiate relationships, and actively contribute to knowledge production (Allen, 2008). With a focus on agency, we refer to the different ways and capacities in which forced migrants actively engage with, make sense of, and respond to their experiences, rather than merely being passively subjected to them (Ongwech et al., 2024). Existing literature focuses on agency-related themes, including residency, political belonging, transnationalism, and citizenship (Nyers, 2003). Mainwaring (2016) argues that the most common approach to migrant agency studies focuses on instances of resistance and transgression on the part of migrants (Nyers, 2003), with a less common approach examining the everyday forms of agency and empowerment, such as how migrants and refugees adapt daily routines in the face of their legal status.

Researchers in migration and mobility studies have begun utilizing creative and alternative approaches to conceptualizing and designing how research practice can respond and adapt to the importance of participants’ agency. Hugman et al. (2011) argue that participatory methods should enhance refugee agency throughout the research process. This involves acknowledging the inherent power imbalances between researchers and refugees while ensuring that the contributions of all participants are equally valued. There is a growing effort to adapt methodologies that more effectively capture the complexity and multifaceted realities of refugees’ lives, both past and present. This shift reflects an increasing appreciation for approaches that align more closely with research questions to explore the nuanced and intricate aspects of human experience (Hugman et al., 2011). The goal is to find more ethical and empowering research for participants in which narratives, findings, and stories are better connected to the voices of the interlocutors and challenge traditional research methodologies to find less extractive approaches that result in benefits for community participants.

Participatory approaches like photovoice and videovoice emphasize participants’ agency by aligning with their lived realities (Lenette et al., 2020). These methods also create spaces to challenge existing power dynamics (O’Reilly, 2020) and foster agency through visibility (Haile, 2020). Refugees and migrants, especially women and LGBTQ+ individuals, are often reduced to passive, dehumanized statistics (Fiddian-Qasmiyeh, 2016). Compared to other methods, photovoice and videovoice provide participants with greater control, reducing the risk of misrepresenting their ideas (Lenette, 2020). Few studies have explored how migrants and refugees can exercise their agency through visibility and self-representation using participant-driven photography or video.

Recent scholarship has expanded participatory visual research by integrating digital and social media platforms, particularly Instagram, into photovoice and videovoice methodologies. Early participatory frameworks emphasized empowerment through image-making and collective reflection (Wang & Burris, 1997), while newer studies highlight how digital tools extend this practice into online and transnational spaces (Gubrium & Harper, 2016; Latz & Mulvihill, 2017). Researchers have argued that Instagram’s aesthetic culture and interactive affordances enable participants to engage in visual storytelling that circulates beyond the confines of traditional research dissemination (Leaver et al., 2020). Latz and Mulvihill (2017, p. 141) get at the close link between Instagram and photovoice by asking: “Is Instagram the world’s largest photovoice project?” The platform’s immediacy and participatory dynamics foster agency and visibility, especially for marginalized groups, but also raise new ethical and methodological concerns regarding consent, surveillance, and digital permanence (Chouliaraki & Georgiou, 2022; Rose, 2016). Consequently, contemporary scholars increasingly view Instagram not merely as a dissemination tool but as a methodological environment that reshapes how photovoice and videovoice can capture, contest, and share lived experiences in digitally mediated contexts (Jarldorn, 2018).

## The Project

The project engaged LGBTQ+ refugees and migrants living in São Paulo to explore various social identities and enhance migrant and refugee agency by investigating mobility through arts-based methodologies, reflective practices, and participant-centered advocacy. Ultimately, this project aimed to document the lived experiences of LGBTQ+ refugees and migrants in Brazil, addressing a significant gap in research on queer migration, which typically focuses on South-North migration, ignoring South-South migration.

The idea for this project grew out of a partnership with the LGBTQ+ refugee shelter Casa Miga and the NGO Manifesta LGBT+, in Manaus, Brazil, which began in 2019. Through two earlier research projects with Casa Miga, we heard directly from research participants that the pandemic left them feeling “frozen” and “voiceless.” The research team realized that creative approaches were needed to better capture LGBTQ+ refugees’ and migrants’ experiences and give them more agency to share their stories on their own terms. We therefore co-designed and iteratively refined the study with frontline partners in São Paulo and Manaus. Casa Miga, Manifesta LGBT+, Caritas Brasileira, and local LGBT activists and community leaders supported us in piloting the photovoice and videovoice activities. Over 3 months, three groups of five LGBTQ+ migrants tested the instruments and activities, with feedback collected after each round. This feedback guided refinements to the methods, such as hosting workshops in LGBTQ+-affirming venues and tailoring content after each cohort to ensure the workshops met participants’ needs. The piloting also led us to select Instagram as the primary platform for capturing and editing photos and videos. Participants felt Facebook and Twitter were less safe or suitable for LGBTQ+ self-expression, while Instagram offered features that aligned with their interests, particularly the ability to create and edit short reels, and was the platform they felt most comfortable using.

Participant recruitment and engagement were carried out through snowball sampling via local NGOs and refugee shelters in São Paulo, with workshop logistics and compensation designed collaboratively to ensure fairness and feasibility. A local LGBTQ+ community leader was hired as field coordinator, helping establish trust and recruit 20 participants from Venezuela, Colombia, Mozambique, Haiti, and Guinea-Bissau. The participants in our project come from various social and economic backgrounds, from different migration contexts with diverse social identities.

Workshops were held in LGBTQ+ friendly venues that were chosen for accessibility and safety. Psychological and digital safety were prioritized through icebreakers, open communication, and training on privacy and online risk mitigation, with participants assured of confidentiality and their right to withdraw without penalty. The accessible and LGBTQ+-friendly space ensured that they were in a space that would make them feel welcomed, and the training helped to educate them on how to protect themselves from potential harm on digital platforms. Across three participatory workshops held over 7 months in 2023, participants received hands-on training in Instagram storytelling and video editing using tools such as CapCut. Peer-to-peer learning bridged digital literacy gaps and strengthened community bonds, culminating in certificates of completion to recognize participants’ contributions. WhatsApp groups, a preferred mode of communication in Brazil, were formed for participants for prompts, check-ins, and peer support. Each participant received R$300 (USD $60) in two installments to cover transport, meals, and data costs. Workshop One introduced project goals, ethics, and basic media skills. Then, participants were asked to spend two weeks making content about their lives in São Paulo. Then they came back together during Workshop Two to share their content and reflect together as a group on recurring themes such as discrimination, belonging, and public perceptions of LGBTQ+ migrants. The project concluded with a curated video compilation of participant-generated content, showcasing the diversity and depth of their experiences. In Workshop Two, a common comment among participants was the diversity of their experiences, despite all being LGBTQ+ migrants and refugees in the same city. As such, we decided that in addition to their individual photos and videos, we should also have a compilation of them and have it uploaded on YouTube so others can see it. This video was presented at conferences in London, Toronto, Oxford, and Twente. The compilation amplified marginalized voices and demonstrated how participatory visual methods can foster agency and disrupt traditional researcher-participant power dynamics.

### Instagram and Ethical Considerations

The workshops emphasized safety and respect for others: we walked through privacy settings, when not to film/photograph, and the permissions needed if they wanted to feature other people’s faces in their content. The fieldwork coordinator used WhatsApp to reinforce safe-use reminders during the time participants were making content.

Questions of ethics were addressed head-on and repeatedly. Consent was obtained at every step (initial survey, training workshop, content creation time, and final workshop), with the right to withdraw and keep honoraria; media permissions were granular in our informed consent form (audio, video, and photos). Because Instagram/WhatsApp are external platforms, consent materials explicitly warned about platform surveillance/third-party access. Visual data from photovoice and videovoice were not confidential by default, and this was made clear in the informed consent form. However, all participants consented to this and were open about wanting to be visible and take ownership of their own stories. Everyone in the workshops was comfortable with being visible and sharing photos of themselves and their lives. The authors recognize the recruitment bias that influenced participant engagement in the project, because if you were uncomfortable with the lack of confidentiality, then you would be excluded from participating. Therefore, it is not representative of all LGBTQI+ migrant and refugee voices in Brazil.

## Results and Discussion: Participant Experiences and Intersectional Storytelling

The outcomes of this project reveal both the possibilities and the constraints of using Instagram as a tool for data gathering in participatory visual methods with LGBTQ+ refugees and migrants. We adopted a participatory interpretive approach (Redman-MacLaren et al., 2014) to reduce power imbalances where participants collaborated with the researchers to present and explain their videos. In final workshops, participants explained the content they created, the stories they sought to tell, and their choices in photos, videos, and edits. Follow-up interviews explored their reflections on the workshops, creative methods, and outcomes. This minimized interpretative bias and center participants’ own voices, ensuring their intentions were accurately understood and represented.

### Engagement, Feasibility, and Early Impressions

All participants completed the workshops and produced between 4 and 10 pieces of content each (including photos and videos), showcasing the feasibility and appeal of Instagram-enabled photovoice and videovoice methods. Many expressed joy at documenting and sharing aspects of their lives often denied to LGBTQ+ people in their countries of origin. As Binta, a young Lesbian Migrant from Mozambique, expressed, “I felt very free, happy, trying to express my life . . . because I could not express so much in my country. It is very good and important too.”

Participants used the project to showcase triumphs, struggles, and everyday life, strengthening their ability to craft life narratives and move beyond reductive labels like “refugee” or “migrant.” Jordan, a gay participant from Venezuela, explained,

It is very important to say that the content that I did is related to clothing, sewing, modeling, and everything I learned here in Brazil. Here is content that reflects what I learned living here in São Paulo, or what I managed to do to evolve as a person, as an immigrant and as part of the community.

For Jordan, the exercise allowed them to showcase their multidimensional selves.

The research approach helped humanize LGBTQ+ migrants by highlighting their struggles, aspirations, and everyday practices, offering a more nuanced and intersectional representation of migrant lives. Angelica, a lesbian participant from Venezuela who has been living in São Paulo for three years, reflected:

As for me, my story was a bit sad because I come from a country where there’s a lot of discrimination. Gay people are discriminated against, and our orientation isn’t accepted, which makes things very complicated. I shared a bit of that story and many others I experienced. This project taught me to keep moving forward, open my eyes more, understand life better, and truly live because we must live our lives.

Participants’ narratives revealed how sexuality, gender, nationality, class, and ethnicity intersected to shape their experiences of displacement and belonging. Cholita, a Bolivian drag performer and entrepreneur emphasized the collaborative dimension of the workshops:

My participation in the project made me feel like it was a real exchange of experiences. We learn from each other’s lives and interactions. It was an exchange of experiences in a different country, with different ethnicities, challenges, and life experiences. It was fascinating in that sense.

These exchanges were drawn out during the debrief workshops, where participants reflected on both the commonalities and divergences in their stories. This process underscored the value of participatory visual methods in interrogating homogenizing narratives, while also demonstrating how an intersectional lens can illuminate the overlapping power structures and identities shaping queer South–South migration.

### Fostering Community in Digital Spaces through Photovoice and Videovoice Research

The project played a pivotal role in community building among LGBTQ+ migrants, particularly in São Paulo, where isolation and loneliness are prevalent. Sharing personal stories created a sense of inclusion and belonging, allowing participants to connect deeply with others who share similar realities and challenges. Ringo, a gay participant from Bolivia, shared,

It was a very cool experience to work with this group of people because I managed to experience and see firsthand the experiences of other immigrants who were also aware that it was difficult, it was complicated because leaving home and going to a new country, together with a new language, it’s a challenge.

The workshops and project provided a safe space for participants to share their challenges and vulnerabilities, which is something rarely afforded in a sprawling mega-city like São Paulo. The city’s geographic layout, with many migrants living on its periphery, often deters community building.

Oftentimes, LGBTQ+ migrants face challenges when they first arrive, and those opportunities to build community through these workshops and to express their social and physical mobility fostered personal growth, connection, and a stronger sense of belonging among participants. As Jordan added,

these are vulnerable people, immigrants, part of the community, who need a little more attention. You have people who have had a very difficult time in our first days, in our first months. And showing day by day, showing the qualities, what we do here in the city, not the country, is a very good idea.

Friendships formed during the workshops extended beyond the project’s duration, with participants organizing social gatherings and maintaining vibrant discussions in WhatsApp groups (Alencar & Camargo, 2022). These interactions facilitated a supportive network that bridged the gap between isolation and connection. Many participants expressed that these relationships were among the most meaningful outcomes of the project, and the shared videos and photos deepened their understanding and strengthened bonds within the group. Chiki, a trans participant from Venezuela, described that “those days were wonderful for us. We shared so much, laughed, talked, and spent time together. The experience was beautiful—very beautiful.” In addition, Brain, a participant from Bolivia, explained,

My participation in the project made me feel like it was a real exchange of experiences and skills, you know? We learn from each other’s lives and interactions, so it was interesting. It was an exchange of experiences in a different country, with different ethnicities, challenges, and life experiences. It was fascinating in that sense.

Having a safe space for participants to share their vulnerabilities and connect with others who shared similar experiences as LGBTQI+ migrants living in Sao Paulo helped build community and collective agency. By fostering enduring friendships and support networks, the workshops created reciprocal benefits that are less extractive and more ethical. Participants referenced the lasting friendships they had made from participating in the workshop and were grateful for a collective space in which queer migrants living in the periphery of the city could gather. The formation of these enduring friendships and support networks highlights the power of participatory creative methods in building community and enhancing collective agency. Participants’ willingness to collaborate, mentor, and share ideas, experiences, and technical skills around video editing created a sense of solidarity and empowerment. The newfound skills participants enhanced or developed left people feeling empowered, building a greater sense of agency.

### Instagram as a data collection tool

Using Instagram as the primary tool for capture and caption positioned photovoice and videovoice within participants’ everyday practices of self-expression, advancing the core goal of centering participant voice and control (Pink et al., 2016; Wang & Burris, 1997). Rather than relying on researcher-controlled equipment, participants used a familiar platform to record, edit, sequence, and narrate their experiences in their own voices (Bucher & Helmond, 2018; Leaver et al., 2020). Allowing them to apply filters, stickers, text, and music provided greater creative freedom than traditional photovoice methods with physical cameras. Instagram was therefore not just a conduit but a central part of the research process, aligning data creation with the everyday social and technical practices participants use to negotiate identity, belonging, and visibility (Bucher & Helmond, 2018; Pink et al., 2016).

Methodologically, Instagram shifted visual and narrative control into participants’ hands and removed the researcher as a middle person, reducing logistical and creative interference (Manovich, 2017; Wang & Burris, 1997). It’s built-in tools enabled editing and reflexive captioning, while likes and comments supported ongoing dialogue beyond time-bound workshops (Pickering et al., 2022). Brian, for example, was able to showcase his wig-making business and her drag persona, engaging with potential clients and booking drag performances in the comments. Empirical studies show that fusing photovoice with social media platforms increased engagement and immediacy, amplifying participatory depth when creation and conversation happen in the same familiar environment (Pickering et al., 2022).

For LGBTQ+ refugees and migrants in our project, Instagram’s affordances are mapped onto strategic disclosure and safety management. Participants used pseudonymous accounts, calibrated visibility through privacy controls, and selectively shared their content, all while crafting identity work on their own terms (Craig & McInroy, 2014; Leaver et al., 2020). Recognizing that immigrant creators’ visibility is shaped by recommender systems, our training incorporated “visibility literacy” (captions, language choice, hashtags, audio use) balanced with safety practices (pseudonyms, Close Friends, archive/private posts), adapting insights from other social researchers (Divon et al., 2025; Jaramillo-Dent et al., 2022).

At the same time, Instagram revealed the ambivalent nature of digital agency. While the platform offered stylistic and narrative control, allowing participants to decide how much to disclose, which audiences to engage, and how to curate their stories, it also exposed them to vulnerabilities tied to platform surveillance, algorithmic invisibility, and the risks of digital circulation beyond their control (Divon et al., 2025; Jaramillo-Dent et al., 2022). Participants managed these risks through privacy settings and pseudonyms, yet these strategies underscore the limits of digital autonomy in platform-mediated spaces.

Occasionally, autonomy was further constrained by technical barriers that required research assistance. This tension between independence and facilitation underscores the ongoing challenge of balancing empowerment with practical support in participatory digital research. As Chiki reflected,

I felt very good because I’m not someone who’s used to being in front of a camera or making videos. I don’t feel very comfortable with cameras, but in the end, I succeeded because when you set your mind to something, you achieve it. My experience was wonderful because I learned a lot. I learned that each person has their own story and that we all go through a process.

Similarly, Ringo explained, “I made videos, I really like talking in videos about good projects, each person’s life, my work, how I am here.” These reflections illustrate how Instagram simultaneously empowered participants to claim visibility and connection, even as their agency remained negotiated within the platform’s structural constraints.

### Agency Through Creative Expression and Intersectional Storytelling

Photovoice and videovoice gave participants flexibility to present their authentic daily lives, perform and experiment with identity, or maintain familiar modes of self-representation. We intentionally avoided prescribing topics, themes, or questions, allowing participants to decide how to use the medium. This was particularly salient for Venezuelan LGBTQ+ asylum seekers in Manaus during the pandemic, who reported feeling “frozen” in time and status, encountering discrimination and exclusion in work and housing due to racism, xenophobia, homophobia, and transphobia (Cowper-Smith et al., 2021; Su, 2023; Su et al., 2021). Creative methods enable participants to reclaim narrative control and challenge oppressive regimes, yet agency remained uneven: fears around visibility, unequal device access/skills, and precarity, limited some participants’ capacity to engage fully. Lina, a 55-year-old participant from Haiti, was familiar with Instagram but was not familiar with photo and video editing tools. As a result, she relied heavily on her peers to support her in the editing process. As Lenette (2020) and O’Reilly (2020) remind us, participatory projects do not automatically equalize power relations; rather, agency is continuously negotiated within the boundaries set by structural inequalities and research design. Recognizing these constraints is essential to avoid overstating the liberatory potential of creative methodologies.

Bringing photovoice and videovoice together over Instagram helped mitigate existing power dynamics by aligning the method with their lived realities (Lenette et al., 2020; O’Reilly, 2020). These methods provided participants with opportunities to exercise power, negotiate relationships, and actively contribute to knowledge production. For instance, Binta’s reflection on expressing her life through creative means highlights the potential of such methods and the ability of these creative methods to foster more agency. Moreover, the exchange of technical skills between participants in photo and video editing enhanced confidence, allowing participants to enrich their storytelling capabilities and feel a sense of pride in their newly developed skills. The collaborative learning environments created through the workshops and through bringing people together exemplify everyday forms of agency through collective empowerment. However, participation required familiarity with Instagram and a willingness to engage with the platform, which shaped who could participate and the forms of agency available.

Centering South-South stories disrupted dominant narratives that cast LGBQT+ mobility as a one-way movement from “oppressive” South to “liberated” global North (Valiquette et al., 2021), and resisted damage-centered representations (Tuck, 2009) and the “good/bad” migrant dichotomy (Fiddian-Qasmiyeh, 2014). The possibility of telling their own stories in their own terms allowed them to explore their migration journeys beyond dominant narratives. This included both their physical mobility and their social adaptation to life in São Paulo. For example, Jordan’s storytelling revealed his personal growth and integration into the community, showcasing what Mainwaring (2016) describes as migrants’ everyday forms of empowerment in adapting to new environments. As Brian’s previous example with wig making and selling highlights, the creative outputs reflected participants’ abilities to navigate challenges and leverage opportunities, emphasizing their adaptability and resilience. This approach aligns with Lenette’s (2020) argument that participatory creative methods allow migrants to take ownership of their narratives and reduce the risk of misrepresentation.

Moreover, an intersectional approach emphasizes the importance of analyzing multiple and interconnected social categories rather than treating identities as independent (Fiddian-Qasmiyeh, 2020). Photovoice and videovoice created opportunities for participants to represent themselves in ways that, when analyzed through an intersectional lens, foregrounded the diversity and complexity of their lives. Participatory and creative methodologies like these empower participants to contribute to the research process actively, ensuring the focus aligns with their priorities while uncovering nuanced aspects of their lives (Teti et al., 2021). This approach is especially relevant to uncovering the diversity of experiences among LGBTQI+ migrants and refugees rather than assuming a homogeneity of experiences, which can occur in research on migration and mobility. The use of video allowed for richer material that, when interpreted through an intersectional lens, illuminated not only participants’ chosen narratives but also contextual elements such as class, space, and community. Angie’s video highlighted her social networks with other Venezuelans, the apartment and space they shared, and specific Venezuelan Spanish slang. Their video showed their everyday life, which included cooking meals with her friends in their small apartment and playing with her two dogs.

Video’s motion, sound, and narration often provided richer intersectional material than static images (Catalani et al., 2012; Teti et al., 2021). Selfie videos captured accent, mobility, and context; participants spoke directly to audiences, highlighting chosen priorities. In two-minute Instagram videos, participants showcased work, socializing, education, religion, gender expression, and advocacy. For example, Cholita represented her playful, creative drag identity while also showing her work and social position as a migrant, trans woman, and professional performer. Brian combined his identity as an LGBTQI+ migrant with his craft as a wig maker in São Paulo. Roccio, a Peruvian participant, highlighted their intersectional identities as a student, professional, and lesbian migrant, resisting being seen as solely a lesbian migrant. Nikita from Tunisia celebrated their trans identity, culture, and mobility in their content by incorporating Tunisian music and mapping life in and around São Paulo. These multilayered narratives complicate homogeneous portrayals and surface tangible points for intervention (Fiddian-Qasmiyeh, 2020; Teti et al., 2021).

However, it is important to note the limits of representing intersectional experiences through visual methods. While photos and videos can surface multiple facets of identity, not all dimensions of inequality are equally visible, and filming and editing choices may foreground some aspects while obscuring others. For example, Binta from Mozambique chose to share videos of their social life mainly through selfie-style photos and videos, which don’t give the viewer much context for their surroundings, with the focus on the fun they are having. Given the challenges that LGBTQ+ people face in Brazil, it is conceivable that Binta would also experience discrimination, harassment and other issues in their social life that is not shown in their videos. Moreover, structural constraints, such as platform policies, researcher facilitation, and participants’ differing levels of digital literacy, shaped what could and could not be expressed. Recognizing these limits prevents an overly celebratory view of participatory visual methods and reinforces the importance of critical reflexivity when applying an intersectional lens.

In addition, when groups returned to share their videos, we engaged in collective analysis by asking participants to reflect on both what they had chosen to share intentionally and what might have been conveyed unintentionally. The debrief sessions were guided by an intersectional lens, encouraging discussion of how the videos captured multiple, overlapping aspects of identity and experience. At the same time, even with an intersectional framing, not all dynamics of inequality or identity were easily articulated, and researcher facilitation inevitably shaped which reflections were emphasized.

Finally, a video’s ability to capture subtle markers such as accents, mobility, and geographic context ensures that participants’ intersectional experiences are represented in ways that static methods might overlook. Methodologically, this adds depth to the qualitative analysis by revealing details that may otherwise go unnoticed. This aligns with intersectionality’s usefulness in addressing the interconnected nature of social categories and power structures (Lennet, 2020; Romero, 2017). By combining photovoice’s visual emphasis with videovoice’s dynamic storytelling, this combined creative methodology not only empowers participants but also provides richer, more nuanced data for researchers. When examined through an intersectional framework, the data advances knowledge and practice in more equitable ways, aligning with the goals of participatory visual research and demonstrating how intersectionality as an analytical framework can deepen understandings of queer migration, displacement, and mobility.

## Conclusion: Reframing Queer Migration Through Digital Participatory Methods

This study demonstrates the use of Instagram as a tool for photovoice and videovoice as forms of participatory digital storytelling to amplify the voices and agency of LGBTQ+ refugees and migrants in São Paulo, Brazil. By centering participants as co-creators of knowledge and empowering them to narrate their migration journeys on their own terms, this research challenges dominant narratives that often homogenize or reduce queer migrants to passive subjects of study. Instead, it highlights their creativity, resilience, and capacity for self-representation, disrupting power imbalances inherent in traditional research methodologies.

The innovative use of Instagram and phone-based editing tools as mediums for photovoice and videovoice not only resonates with participants’ existing digital practices but also facilitates more inclusive and accessible storytelling. Participants exercised stylistic and narrative control by writing captions, deploying filters and music, and calibrating visibility through privacy settings. This integration of creation, curation, and circulation within a single familiar environment proved especially effective for participants managing issues of safety, disclosure, and community connection. This approach allowed participants to represent their multifaceted identities, whether as students, workers, artists, or community advocates, while embracing their everyday struggles and triumphs. By adopting an intersectional lens, the study became more sensitive to the overlapping inequalities participants navigated, ensuring that their creativity and agency were understood in relation to gender, sexuality, race, and migration status. By creating space for free-thinking and agency, participants could transcend imposed labels like “migrant,” “refugee,” or “outsider” and reclaim their narratives as individuals with complex, intersecting identities.

The findings underscore the importance of participatory creative methods in advancing intersectional approaches to migration research. Participants’ diverse content, ranging from professional aspirations to cultural pride and community building, revealed how mobility is shaped by interconnected social identities and power structures, including gender, race, sexuality, and socioeconomic status. Through this dynamic process, photovoice and videovoice proved particularly effective in capturing the nuances of these identities and highlighting intragroup diversity that static or prescriptive methods might overlook. However, several structural and ethical challenges remained. Platform policies, researcher facilitation, and varying digital literacy shaped what could be expressed, while not all inequalities were easily articulated. The use of social media also introduced risks of surveillance, algorithmic invisibility, and limited control over digital circulation, exposing the tensions inherent in participatory online research.

Moreover, the project’s emphasis on community building reinforced the collective agency of LGBTQ+ refugees and migrants, fostering lasting relationships and solidarity within a population often marginalized and isolated. By creating a safe, collaborative, and empowering space, this research demonstrates how participatory methods can drive not only knowledge production but also social mobilization, advocacy, and meaningful change.

In conclusion, flipping the lens through photovoice and videovoice reveals the critical role of creativity, agency, and intersectionality in migration scholarship. Our integration of Instagram as a data collection tool in traditional participatory methods marks a distinctive contribution, showing how digital platforms familiar to participants can strengthen agency, reduce researcher intervention, and extend participatory dialogue beyond bounded research encounters By empowering participants to show and tell their stories, this study challenges normative depictions of migration, reframes marginalized experiences, and highlights the potential of participatory methodologies to produce equitable and nuanced research. Future studies and policy interventions must continue to prioritize these creative approaches to ensure that the voices and lived realities of LGBTQ+ migrants and refugees are recognized, valued, and heard on a global stage.
